# Supplementary material for: Unravelling reference bias in ancient DNA datasets
Source: Bioinformatics. 2024 Jul 3;40(7):btae436. doi: 10.1093/bioinformatics/btae436 (PMC11254355; doi:10.1093/bioinformatics/btae436)
Supplement: btae436_Supplementary_Data [file btae436_supplementary_data.zip › S1-S4_data_plots.zip!/S1-S4_data/rhino_sim_plots.zip!/rhino_sim_plots/local_bowtie_rhino_div5_10end_by_MQ.pdf]

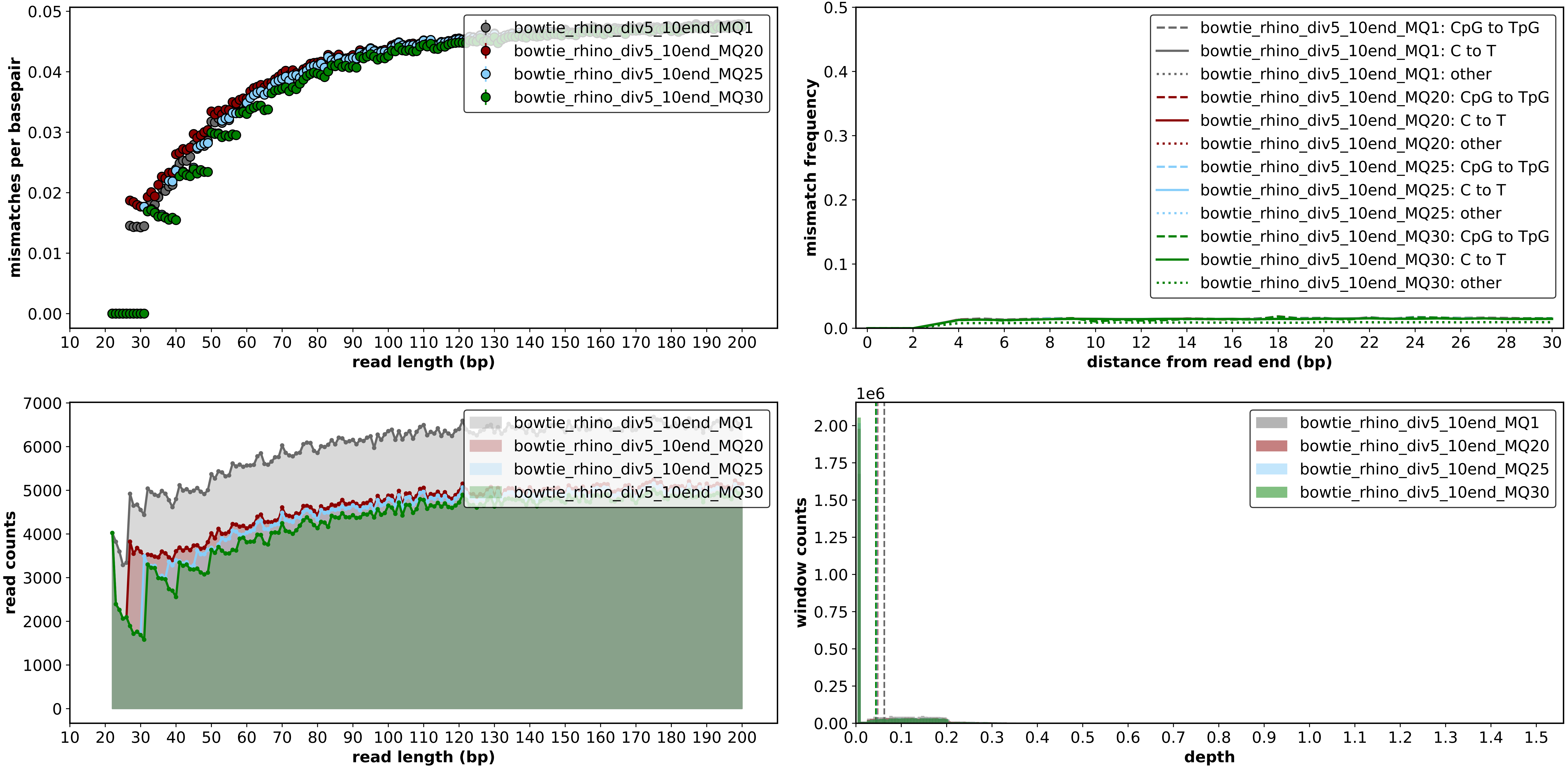
mismatches per basepair
0.05
0.04
0.03
0.02
0.01
0.00
read length (bp)
10 20 30 40 50 60 70 80 90 100 110 120 130 140 150 160 170 180 190 200
bowtie_rhino_div5_10end_MQ1
bowtie_rhino_div5_10end_MQ20
bowtie_rhino_div5_10end_MQ25
bowtie_rhino_div5_10end_MQ30
mismatch frequency
0.5
0.4
0.3
0.2
0.1
0.0
distance from read end (bp)
0 2 4 6 8 10 12 14 16 18 20 22 24 26 28 30
bowtie_rhino_div5_10end_MQ1: CpG to TpG
bowtie_rhino_div5_10end_MQ1: C to T
bowtie_rhino_div5_10end_MQ1: other
bowtie_rhino_div5_10end_MQ20: CpG to TpG
bowtie_rhino_div5_10end_MQ20: C to T
bowtie_rhino_div5_10end_MQ20: other
bowtie_rhino_div5_10end_MQ25: CpG to TpG
bowtie_rhino_div5_10end_MQ25: C to T
bowtie_rhino_div5_10end_MQ25: other
bowtie_rhino_div5_10end_MQ30: CpG to TpG
bowtie_rhino_div5_10end_MQ30: C to T
bowtie_rhino_div5_10end_MQ30: other
read counts
7000
6000
5000
4000
3000
2000
1000
0
read length (bp)
10 20 30 40 50 60 70 80 90 100 110 120 130 140 150 160 170 180 190 200
bowtie_rhino_div5_10end_MQ1
bowtie_rhino_div5_10end_MQ20
bowtie_rhino_div5_10end_MQ25
bowtie_rhino_div5_10end_MQ30
1e6
window counts
2.00
1.75
1.50
1.25
1.00
0.75
0.50
0.25
0.00
depth
0.0 0.1 0.2 0.3 0.4 0.5 0.6 0.7 0.8 0.9 1.0 1.1 1.2 1.3 1.4 1.5
bowtie_rhino_div5_10end_MQ1
bowtie_rhino_div5_10end_MQ20
bowtie_rhino_div5_10end_MQ25
bowtie_rhino_div5_10end_MQ30
